# Safety Assessment of *Acer tegmentosum* Maxim. Water Extract: General Toxicity Studies in Sprague–Dawley Rats and Beagle Dogs With Re-evaluation of Genotoxic Potentials

**DOI:** 10.3389/fphar.2021.687261

**Published:** 2021-08-31

**Authors:** Jin-Sung Park, Euna Kwon, Yun-Soon Kim, Sang-Moo Kim, Dae-Sun Kim, Ja-June Jang, Jun-Won Yun, Byeong-Cheol Kang

**Affiliations:** ^1^ Department of Experimental Animal Research, Biomedical Research Institute, Seoul National University Hospital, Seoul, South Korea; ^2^ Graduate School of Translational Medicine, Seoul National University College of Medicine, Seoul, South Korea; ^3^ Pharm Cross., Ltd, Chuncheon, South Korea; ^4^ Department of Pathology, Seoul National University College of Medicine, Seoul, South Korea; ^5^ Department of Biotechnology, The Catholic University of Korea, Bucheon, South Korea; ^6^ Biomedical Center for Animal Resource and Development, Seoul National University College of Medicine, Seoul, South Korea; ^7^ Designed Animal and Transplantation Research Institute, Institute of GreenBio Science Technology, Seoul National University, Pyeongchang-gun, South Korea

**Keywords:** *Acer tegmentosum* Maxim., salidroside, repeated oral toxicity, rodents, beagle dogs, genotoxicity

## Abstract

*Acer tegmentosum* Maxim., commonly known as Manchurian stripe maple, is a deciduous tree belonging to the family of Aceraceae and has been traditionally used in folk medicine for its remedial effects in liver diseases and traumatic bleedings. With a growing body of experimental evidence for its pharmacological efficacies, such as neuroprotective, hepatoprotective, antioxidant, and anti-inflammatory activities, *A. tegmentosum* has gradually gained popularity as a health supplement and functional food. However, the large part of essential toxicity information still remained lacking despite the possibility of mutagenic potentials as previously suggested, posing safety concerns for human consumption. In this study, we evaluated 90-day repeated oral toxicity of *A. tegmentosum* Maxim. water extract (ATWE) in SD rats with acute toxicity assessment in beagle dogs, and reevaluated genotoxicity using a combination of *in vitro* and *in vivo* assays. During the oral study period, ATWE did not cause toxicity-related clinical signs and mortality in rodents without adverse effects observed in the analysis of hematology, serum biochemistry, and histopathology, establishing >5,000 mg/kg BW as the NOAEL. In addition, doses up to 5,000 mg/kg BW did not cause acute toxicity in beagle dogs. When assessed for genotoxicity using bacterial reverse mutation, chromosome aberration, and micronucleus formation, ATWE showed lack of mutagenicity and clastogenicity. These results demonstrated that AWTE was safe in the present preclinical study for systemic toxicity and genotoxicity at the tested doses, providing a guideline for safe use in humans.

## Introduction

The family of Aceraceae, also known as the maple family, is a group of deciduous trees and shrubs which contains 156 species (Cambelbeke, 2020). Among them, about 40 species have been used for medicinal purpose in the world (Bi et al., 2016). *Acer tegmentosum* Maxim. is commonly known as Manchurian stripe maple, and extracts of its parts, including the boughs and twigs, have been a popular ingredient in traditional medicine based on its empirical effects to treat various diseases such as liver diseases and traumatic bleeding. Recent studies reported that extracts from *A. tegmentosum* possessed various therapeutic effects in *in vitro* and *in vivo* model systems, including hepatoprotection (Park et al., 2019), alleviation of alcohol-induced liver injury and steatosis (Lee Y. et al., 2017), antioxidant and anti-inflammation (Kim et al., 2012), suppression of atopic dermatitis (Yang et al., 2016), reduction of bone destruction (Ha et al., 2014), and antidepressant activity (Park et al., 2020a).

The complete list of compounds contained in *A. tegmentosum* have not been reported yet, but to date, 44 compounds have been identified (Park et al., 2006; Tung et al., 2008; Kim et al., 2012; Lee et al., 2014; Lee et al., 2017a; Hou et al., 2019; Piao et al., 2020): 24 flavonoids (e.g., quercitrin, catechin, kaempferol-3-rhamnoside, erigeside B, feniculin, and avicularin), 10 phenolic glycosides (e.g., salidroside, 6′-O-galloylsalidroside, and 3,5-dimethoxy-4-hydroxyphenyl-I-O-β-D-glucoside), 3 phenolic acids (gallic acid, methyl gallate, and 3,4-dihydroxybenzoic acid), 2 phenylpropanoid glycosides (eutigoside A and grayanoside A), 2 courmarins (fraxin and scopoletin), 1 quinone (2,6-dimethoxy-ρ-hydroquinone), 1 phenylethyl alcohol (tyrosol), and 1 steroidal glycoside (β-sitosterol 3-O-β-D-glucopyranoside) (the complete list is available in Table 1). Among these, several compounds have been known to provide beneficial effects in human diseases such as cancer, cardiovascular diseases, and neurodegenerative diseases (Nijveldt et al., 2001; Jucá et al., 2020).

**TABLE 1:** Compounds identified in *Acer tegmentosum*.

	Compounds	References
Coumarins		
1	Fraxin	5
2	Scopoletin	3
Flavonoids		
3	(-)-Catechin	5
4	(-)-Epicatechin	4
5	(-)-Epicatechin gallate	1
6	(-)-Epicatechin-3-O-gallate	1
7	(+)-Catechin	4, 7
8	(+)-Catechin-3-O-(3,4-dihydroxybenzoyl)	7
9	3,7,3′,4′-Tetramethyl-quercetin	5
10	5,3′-Dihydroxy-3,7,4′-trimethoxy flavone	5
11	6-Carboxy-(-)-catechin methyl ester	1
12	6-Hydroxy-quercetin-3-O-galactose	7
13	Avicularin	1, 4
14	Dihydromyricetin	7
15	Erigeside B	7
16	Feniculin	4
17	Gallocatechin	1,7
18	Hyperin	7
19	Kaempferol-3-rhamnoside	7
20	Morin-3-O-α-L-lyxoside	5
21	Myricitrin	7
22	Quercetin	2
23	Quercetin 3-O-β-glucopyranoside	2
24	Quercetin-3-O-[β-D-xylopyranosyl-(1→2)-β-glucopyranoside]	7
25	Quercetin-3-O-β-arabinopyranoside	1
26	Quercitrin	7
Phenolic glycosides		
27	1,2,4-Tri-O-galloyl-β-D-glucose	3
28	3,5-Dimethoxy-4-hydroxyphenyl-I-O-β-D-glucoside	5
29	3,5-Dimethyl-benzyl alcohol 4-O-β-D-glucopyranoside	5
30	3′-O-Galloylsalidroside	7
31	4-(2,3-Dihydroxy propyl)-2,6-dimethoxy phenyl β-D-glucopyranoside	5
32	4-Hydroxyphenylethyl-1-O-β-D-[6′-O-(4-hydroxybenzoyl)]-glucopyranoside	6
33	6′-O-Galloylsalidroside	4, 7
34	Phenylethyl-O-β-D-xylopyranosyl-(1→2)-β-D-glucopyranoside	7
35	Salidroside	5, 6, 7
36	ρ-Hydroxy phenylethyl alcohol 1-O-β-D-(6-O-galloyl)-glucopyranoside	6
Phenylpropanoid glycosides		
37	Eutigoside A	6
38	Grayanoside A	6
Phenolic acids		
39	Gallic acid	6
40	Methyl gallate	6
41	3,4-Dihydroxybenzoic acid	6
Phenylethyl alcohol		
42	Tyrosol	3
Quinones		
43	2,6-Dimethoxy-ρ-hydroquinone	5
Steroidal glycoside		
44	β-Sitosterol 3-O-β-D-glucopyranoside	2

1; Hou et al., 2019, 2; Kim et al., 2012, 3; Lee et al., 2017a, 4; Lee et al., 2014, 5; Park et al., 2006, 6; Piao et al., 2020, 7; Tung et al., 2008

Salidroside or rhodioloside, one of the enriched compounds in *A. tegmentosum*, is a phenolic glycoside which has been widely known as one of the major components in *Rhodiola* species and plays a crucial role in the pharmacological effects of these medicinal herbs. Several studies have reported that the compound has neuroprotective (Zhong et al., 2018), anti-inflammatory (Lee et al., 2014), anticancer (Hu et al., 2010), cardioprotective (Zhang et al., 2012; Zhu et al., 2015), hepatoprotective (Song et al., 2003), and antiaging activities (Mao et al., 2010), suggesting its potentials as a multipotent drug (Magani et al., 2020). Consistent with the variety of its effects, salidroside has been shown to regulate multiple molecular targets including neuropeptide metabolism through the inhibition of prolyl endopeptidase (Fan et al., 2001) and autophagy through modulating the mammalian target of rapamycin activity (Liu et al., 2012; Yin et al., 2016).

**FIGURE 1:**
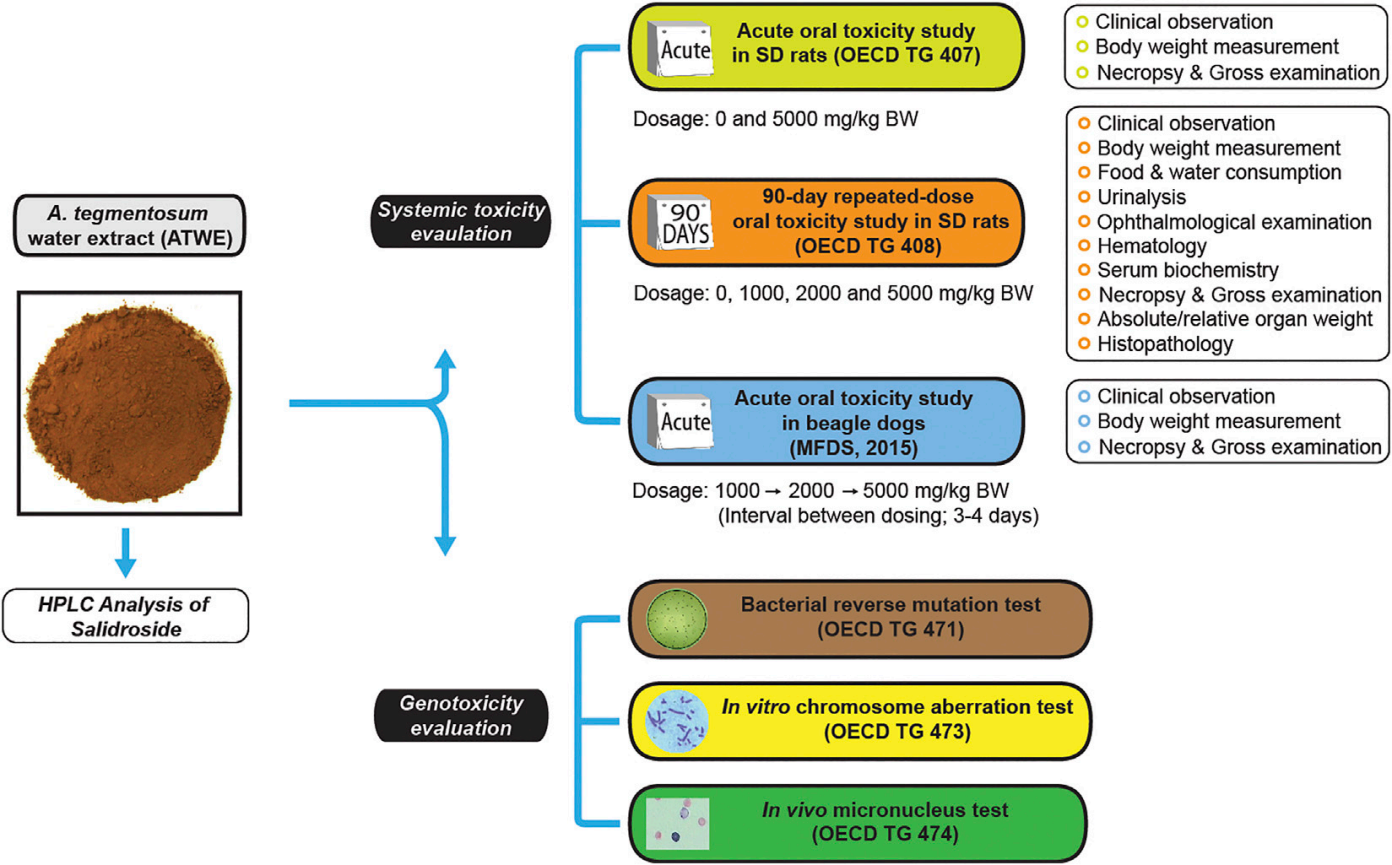
Study design for safety evaluation of *Acer tegmentosum* Maxim. water extract. A schematic drawing shows the study design for systemic toxicity assessment of *A. tegmentosum* water extract (ATWE) from preparation and analysis, general toxicity studies for evaluation of acute and 90-day repeated-dose oral toxicity in SD rats, acute oral toxicity in beagle dogs, and genotoxicity studies for mutagenic (bacterial reverse mutation test) and clastrogenic potential (*in vitro* chromosome aberration test and *in vivo* micronucleus test). The doses and measured parameters are given for each general toxicity study.

The growing body of evidence for health benefits of *A. tegmentosum* and its components has drawn increasing attention of general public, thereby facilitating the development of the *A. tegmentosum*–containing supplements and functional foods as well as their use for the purpose of health promotion. Generally, *A. tegmentosum* has been known for its low risks (Kim et al., 2008) as only mild side effects such as abdominal pain and diarrhea are infrequently observed in cases of overdose, but the recent development and commercialization of highly concentrated *A. tegmentosum* products has greatly increased the possibility of human overdose and manifestation of unidentified toxicity. Despite being known for its safe use, systematic information on the toxicity of *A. tegmentosum* has been largely limited due to the lack of research; to date, only a single study reported the results of an acute toxicity study in rodents (Hwang et al., 2013). Moreover, the bacterial reserve mutation test performed as a part of genotoxicity assessment in the study was inconclusive regarding the mutagenicity of *A. tegmentosum*. However, no further study has been carried out to independently validate the results, posing serious safety concerns on human consumption of *A. tegmentosum* extracts. In this study, we systemically assessed acute and repeated oral toxicity of *A. tegmentosum* Maxim. water extract (ATWE) in SD rats and acute toxicity in beagle dogs (Figure 1). We also reevaluated genotoxicity of ATWE by performing a battery of tests, including the bacterial reverse mutation test, *in vitro* chromosome aberration test, and *in vivo* micronucleus test. Our findings in this study indicate that ATWE was safe in the range of the tested doses in both species and was not mutagenic, providing the essential information and guideline for its safe use in humans.

## Materials and Methods

### Test Substance and Chemicals

*Acer tegmentosum* Maxim. used in this study was collected from Jeongseon area, Gangwon-do, South Korea, and purchased through a local medicinal herb store. The test substance was prepared by a series of processes including extraction from a 1:20 mixture of *A. tegmentosum* Maxim. twigs and tap water at 100°C for 12 h, concentration, filtration, and lyophilization. The resulting test substance ATWE was 74.7 g per kg of the twigs used. Voucher specimens (SNUH 16013-1 and 16013-5) have been deposited at the Department of Experimental Animal Research, Biomedical Research Institute, Seoul National University Hospital. All other chemicals were obtained from Sigma (United States) unless otherwise stated.

### Quantification of Salidroside

The amount of salidroside in ATWE was quantified using HPLC. Briefly, 100 mg of ATWE was dissolved in 25 ml DW under sonication and methanol was added to make it 50 ml. The resulting solution was filtered through a 0.45-µm filter, and 10 µl was separated in a Cadenza CD-C18 (4.6 mm × 300 mm x 5 µm) column (Imtakt, United States) at 25°C in a Waters HPLC system (a 1525 binary pump with a 1500CH column oven and a 2027 auto sampler, Waters, United States) with detection at 220 nm using a Waters 2489 UV detector and the total runtime of 40 min. Gradient elution was performed with DW (A) and a 1:1 mixture of methanol/isopropanol (B) in the following manner (A:B in percentage): 95%: 5% for 0–40 min except for 20%: 80% for 20–25 min. As the standard, 80–200 µg/ml of salidroside in methanol was used.

### Animals

Specific pathogen-free Sprague–Dawley (SD) rats, ICR mice, and conventional beagle dogs were purchased from Orient bio Inc. (Gyeonggi-do, Korea) and housed in environmentally controlled animal rooms designed for each species at the AAALAC International-accredited animal facility (#001160) of Seoul National University Hospital as previously described (Park et al., 2020b). Briefly, each environmental parameter was maintained as follows: temperature at 20–26°C for rodents and 19–28°C for dogs, relative humidity of 50 ± 20%, 12-h light/dark cycle, ventilation 10–15 times/hour, and light intensity 150–300 Lux. Rodents had free access to a γ irradiation-sterilized laboratory diet (Teklad certified irradiated global 18% protein rodent diet, 2918C, Envigo RSM Inc) and autoclaved water, and beagle dogs were fed once a day with a laboratory dog diet (Lab. Canine, 38070, Cargill Agri Purina) with free access to tap water. All experiments were approved by the Institutional Animal Care and Use Committee in Seoul National University Hospital in accordance with the Guide for the Care and Use of Laboratory Animals, 8th edition.

### Acute Oral Toxicity Study in Rats

Six-Week male and female SD rats were acclimated for a week, and only healthy animals (n = 5/gender/group, male; 223.8–254.1 g and female; 175.1–198.4 g) were used in the study. Acute oral toxicity of ATWE was carried out according to the limit test following the OECD Test Guideline No. 420 (OECD, 2001). After random grouping based on body weight according to the stratified continuous randomization method, animals were orally administered either 0 or 5,000 mg/12 ml/kg BW of ATWE in ddH_2_O once and monitored for clinical signs hourly up to 6 h after treatment and then daily for 14 days with a weekly measurement of body weight. After completion of the clinical observation, necropsy was performed on all surviving animals and examined for macroscopic lesions in organs.

### 90-Day Repeated-Dose Toxicity Study in Rats

The 90-day repeated oral toxicity study was performed in compliance with the relevant OECD Test Guideline No. 408 (OECD, 1998). 6-Week male and female SD rats (n = 10/gender/group, male; 226.8–251.9 g and female; 154.7–175.1 g) were divided into four groups and orally treated with one of the doses including 0, 1,000, 2,000, and 5,000 mg/12 ml/kg BW of ATWE for 90 days. During the treatment period, clinical signs were observed daily, while body weight, and food and water consumption were measured weekly. Half of the animals in each group were subjected to ophthalmological examination and urinalysis in the last week of administration. On completion of oral gavage, all surviving animals were deeply anesthetized using isoflurane and euthanized by exsanguination after blood sampling.

### Hematology and Serum Biochemistry

Whole blood sampled in an EDTA tube at necropsy was analyzed for hematological parameters including red blood cell (RBC) count, platelet count, total and differential count of white blood cells (WBCs), hemogloblin, hematocrit, mean corpuscular volume (MCV), mean corpuscular hemoglobin (MCH), and mean corpuscular hemoglobin concentration (MCHC) using an ADVIA 2120i animal blood counter (Siemens Healthcare Diagnostics Ltd., Ireland). Partial thromboplastin time (PT) and activated partial thromboplastin time (aPTT) were measured using an ACL 100 Coagulation analyzer (Instrumentation Laboratory, United States).

Serum separated from coagulated whole blood was analyzed using a Hitachi 7180 Automatic biochemistry analyzer (Hitachi Ltd., Japan) for blood urea nitrogen (BUN), total cholesterol (TC), total protein (TP), total bilirubin (TB), alkaline phosphatase (ALP), aspartate aminotransferase (AST), alanine aminotransferase (ALT), γ-glutamyl transferase (GGT), creatinine, triglycerides (TG), glucose, albumin/globulin ratio, potassium, chlorine, sodium, calcium, and inorganic phosphorus.

### Histopathology

All major organs were in between doses removed immediately after examination for macroscopic lesions and weighed. The organs were fixed by submerging in appropriate fixatives; Davidson solution for the eyes and Harderian glands, Bouin’s solution for testes and the epididymides, and 10% neutral buffered formalin for all the other organs. The femora and nasal cavities were further processed for decalcification. All fixed tissues were paraffinized after processing in a series of graded ethanol and xylene, sectioned into 2–3 µm slices and stained with hematoxylin and eosin. The tissue slices were examined under a bright-field microscope by a pathologist for histopathological analysis.

### Acute Oral Toxicity Study in Beagle Dogs

The study was performed following the Standard for Toxicity Study of Pharmaceuticals (MFDS, 2015) published by the Ministry of Food and Drug Safety, Korea. Beagle dogs (n = 2/gender) were orally given an increasing dose of ATWE, namely 1,000, 2,000, and 5,000 mg/12 mL/kg, with an interval of 3–4 days between doses. Considering the large volume, oral administration was carried out in two separate times (1st administration between 11:00–12:00 and the 2nd between 15:00–16:00) with a half of daily dose. All animals were kept on fasting for 17 h before the 1st administration of the day and observed for 30 min after treatment with monitoring of clinical signs at every hour for 6 h. For the final dosing, animals were additionally monitored daily for 14 days. The body weight of animals was measured a day before administration, and then on days 2, 3, and 7 and at necropsy. On completion of the observation period, necropsy was performed on all animals after euthanasia by exsanguination under deep anesthesia using tiletamine + zolazepam and xylazine.

### Bacterial Reverse Mutation Test

The bacterial reverse mutation test was performed in compliance with the OCED Test guideline No. 471 (OECD, 1997). Briefly, the cytotoxicity of ATWE was examined using the *Salmonella typhimurium* tester strain TA100 to determine the highest treatment dose. Then, four *S. typhimurium* tester strains TA98, TA100, TA1535, and TA1537 and one *E. coli* tester strain WP2(*uvrA*) (MOLTOX, United States) were treated in triplicate with a dose of 0, 312.5, 625, 1,250, 2,500, and 5,000 µg/plate of ATWE with or without S-9 mixture (Oriental Yeast Co. Ltd., Japan). For each treatment condition, an appropriate positive control was used: TA98, 10.0 µg/plate 2-nitrofluorene for TA98, 5.0 µg/plate of sodium azide for TA100, 0.5 µg/plate of mitomycin C for WP2(*uvrA*), 0.5 µg/plate of sodium azide for TA1535, and 80.0 µg/plate of 9-aminoacridine for TA1537. For the groups co-treated with S-9 mixture, 2.0 µg/plate of 2-aminoanthracene for TA98 and TA100 were used and 5.0 µg/plate for the other tester strains. The number of revertant colonies formed in each tester strain plate was counted after incubation at 37°C for 48 h. All experiments using genetically modified bacterial tester strains were approved by the Institutional Biosafety Committee in Seoul National University Hospital.

### *In vitro* Chromosome Aberration Test

The potential of ATWE to cause chromosome aberration was assessed in Chinese hamster lung (CHL) cells according to the OECD Test Guideline No. 473 (OECD, 2014b). After assessing cytotoxicity by calculating relative population doubling in each treatment condition, CHL cells were plated at 5 x 10^5^ in a 25 cm^2^ flask and grown overnight in a 37°C CO_2_ incubator. On the day of treatment, cells were treated with a dose of 0, 125, 250, and 500 µg/ml of ATWE for the 6- and 24-h treatment groups, and 0, 625, 1,250, and 2,500 µg/ml for the 6-h treatment group with metabolic activation using S-9 mixture. As a positive control, 0.1 µg/ml of mitomycin C and 5.0 µg/ml of cyclophosphamide were used for ATWE and ATWE co-treated with S-9 mixture, respectively. The cells were treated with 0.2 µg/ml of colcemid 2 h before completing the total 24-h incubation, and fixed in cold methanol and glacial acetic acid (3:1, v/v) following brief centrifugation and resuspension in 37°C 0.075 M KCl. The cells were then stained with 4% Giemsa solution, and the number of aberrant chromosome-containing cells and the types of chromosome aberration were determined using bright-field microscopy.

### *In vivo* Micronucleus Test

The micronucleus test using mice was carried out in accordance with the OECD Test guideline No. 474 (OECD, 2014a). 8-Week-old male ICR mice were acclimated for a week, and only healthy animals (30.2–33.5 g) were used in the study. Prior to the test, the maximum tolerated dose and sampling time after treatment were determined in animals (n = 3/group). In the main test, four groups of ICR mice were orally administered a dose of 0, 1,000, 2,000, and 5,000 mg/kg BW once a day for four consecutive days with daily monitoring of clinical signs and body weight. A positive control group received intraperitoneal injection of 2 mg/kg BW mitomycin C 24 h before sacrifice. After euthanasia by cervical dislocation, the bone marrow cells were harvested from the femora using FBS, smeared onto two slide glasses per animal and stained with 5% Giemsa solution for 50 min followed by brief treatment in 0.004% citric acid. A total of 4,000 polychromatic erythrocytes (PCEs) per animal (2000 per slide) were examined under a bright-field microscope, and the number of micronucleus-containing polychromatic erythrocytes (MNPCEs) was determined with % PCE [PCE/normochromatic erythrocytes (NCE) + PCE), and % NCE/PCE calculated.

### Statistical Analysis

All values are presented as mean ± standard deviation. The statistical analysis was carried out using a SPSS software (Version 25, IBM) using appropriate methods: Student’s *t*-test for acute toxicity studies, Fisher’s exact test for *in vitro* chromosome aberration test, and the Kruskal–Wallis test followed by *post hoc* Tukey’s HSD multiple comparison test or one-way ANOVA followed by *post hoc* Dunnett’s *t*-test for all the other tests. A *p* value less than 0.05 was considered statistically significant.

## Results

### Quantification of Salidroside in *Acer tegmentosum* Maxim. Water Extract

**FIGURE 2:**
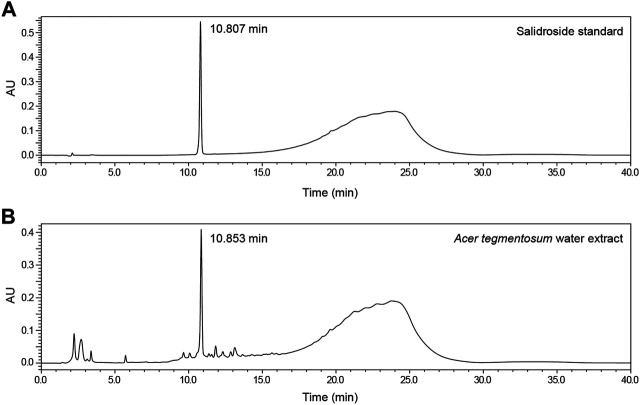
Detection and quantification of salidroside in *Acer tegmentosum* Maxim. water extract. Representative HPLC chromatograms show **(A)** detection of the peak for salidroside at 10.807 min in the standard solution and **(B)** the HPLC profile of *Acer tegmentosum* Maxim. water extract with salidroside detected at 10.853 min (see Materials and Methods for detailed analytical procedures).

*Acer tegmentosum* Maxim. water extract (ATWE) was prepared by performing extraction of dried *A. tegmentosum* twigs in hot water and subsequent lyophilization. Salidroside is a representative pharmacological effector in *A. tegmentosum*, so we selected it as the reference compound and quantified to confirm the validity of ATWE as a test substance for the toxicity study. When measured using HPLC (Figure 2), salidroside was detected around 10.8 min in the chromatogram. The levels of salidroside measured in ATWE were 74.3 ± 0.8 mg/g, indicating that the test substance contained sufficient quantity which showed pharmacological effectiveness in other studies (Hwang et al., 2013; Nugroho et al., 2015; Lee et al., 2017a; Park et al., 2019).

### Acute Oral Toxicity Study in SD Rats

**FIGURE 3:**
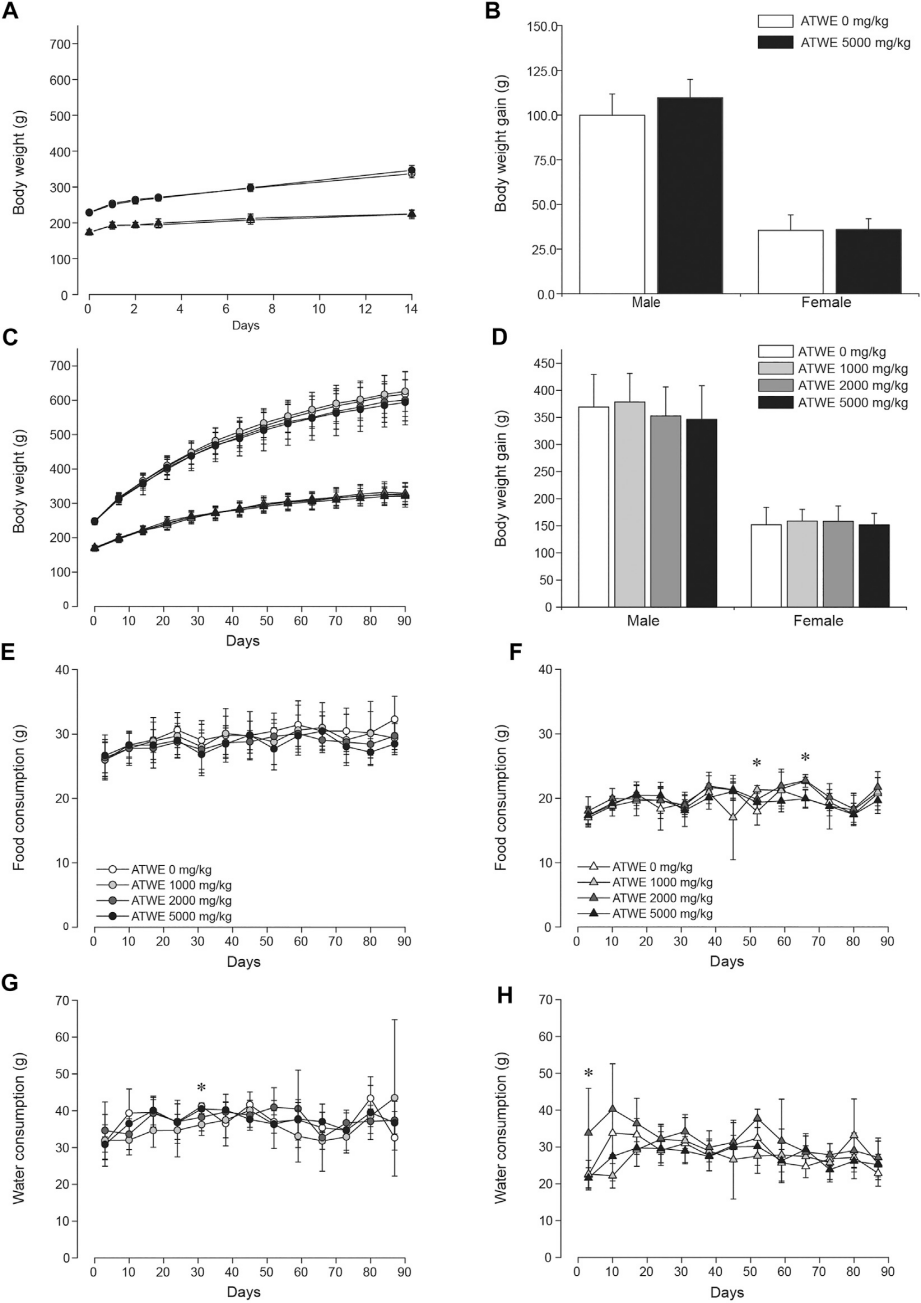
*Acer tegmentosum* Maxim. water extract did not affect body weight gain and dietary patterns in SD rats. Male and female animals were orally administered either 0 or 5,000 mg/kg BW *Acer tegmentosum* Maxim. water extract (ATWE) once and monitored for 14 days in the acute toxicity study (n = 5/gender/group) or one of 0, 1,000, 2,000, and 5,000 mg/kg BW for 90 days in the repeated toxicity study (n = 10/gender/group). In the acute toxicity study, ATWE-treated animals showed a comparable increase of **(A)** body weight for 14 days and **(B)** body weight grain measured at the end of the study to their respective control groups. Similarly, there was no difference in **(C)** body weight and **(D)** body weight gain between the control and AWTE-treated animals regardless of treated doses. The food and water consumption in male **(E, G)** and female **(F, H)** animals was not altered in the test substance–administered groups compared to the control groups. Circles, male; triangle, female; black symbols, 5,000 mg/kg; dark gray symbols, 2,000 mg/kg; light gray symbols, 1,000 mg/kg; and white symbols, 0 mg/kg ATWE. *; *p* < 0.05 by one-way ANOVA followed by *post hoc* Dunnett’s t-test.

Acute oral toxicity was tested in SD rats as a limit test, and considering that the test substance was used as a medicinal ingredient as well as a health supplement, 5,000 mg/kg BW of ATWE was selected for oral administration. None of the male and female animals fed with ATWE showed mortality nor manifested any abnormal clinical signs (data not shown). During the 14-day observation period, ATWE-treated animals showed comparable body weight increase to the control groups (Figure 3A), resulting in a similar weight gain at the end of the study (Figure 3B). In addition, no animals showed toxicity-related pathological lesions at necropsy. These findings indicate that a single administration of 5,000 mg/kg BW was not toxic in SD rats, establishing its LD50 as >5,000 mg/kg BW.

### 90-Day Repeated-Dose Oral Toxicity Study in SD Rats

The acute toxicity study demonstrated that a single oral administration of 5,000 mg/kg BW ATWE was nontoxic in SD rats. Based on these findings, we carried out a subchronic repeated-dose oral toxicity study on ATWE in SD rats; animals were administered a dose of 0, 1,000, 2,000, and 5,000 mg/kg BW once a day for 90 days. We employed three dose levels in this study to ensure that appropriate doses were covered for identification of the no-observable-adverse-effect-level (NOAEL) and target organs due to the potential manifestation of unidentified toxicity in high doses as the test substance has been known to contain many components with limited toxicity information. During the study period, all animals observed were clinically normal with a similar pattern of body weight increase (Figure 3C) and a body weight gain over 90 days (Figure 3D). Consistently, the daily consumption of food and water [(Figure 3E,G for male and Figure 3F,H for female groups] was comparable among all groups with several cases of exception; food consumption was significantly increased in female 100 mg/kg group in week 8, whereas it was reduced in female 1,000 mg/kg and 5,000 mg/kg groups in week 10. When food efficiency was calculated (Supplementary Figure S1), no group showed any remarkable change in these weeks. Water intake was significantly less in male 1,000 mg/kg group (Figure 3G) in week 5 but more in female 2,000 mg/kg group (Figure 3H) in week 1. Despite the detected statistical significance, these findings were considered incidental due to the lack of dose-dependency and a time-dependent tendency of change.

Whole blood collected at necropsy was analyzed for the total RBC and WBC count, different WBC count, and RBC parameters (Table 2). The male 5,000 mg/kg group had a markedly lower WBC count than the control group, but considered insignificant as the value is in our historically normal range. Besides this, all the parameters measured in whole blood showed comparable values across all groups.

**TABLE 2:** Hematological parameters of SD rats orally treated with *Acer tegmentosum* Maxim. water extract for 90 days.

	Dose of *Acer tegmentosum* Maxim. water extract (mg/kg)			
	0	1,000	2,000	5,000
Male (n = 10/group)				
WBC (10^3^/mm^3^)	12.03 ± 3.03	12.20 ± 3.76	10.97 ± 2.33	8.44 ± 1.79^a^
RBC (10^6^/mm^3^)	8.23 ± 0.31	8.27 ± 0.51	8.27 ± 0.36	8.03 ± 0.21
HGB (g/dl)	14.9 ± 0.8	14.5 ± 1.1	14.7 ± 0.6	14.6 ± 0.3
HCT (%)	44.7 ± 2.1	44.7 ± 2.1	44.2 ± 1.9	43.9 ± 0.9
PLT (10^3^/mm^3^)	928.6 ± 89.8	920.9 ± 100	921.7 ± 139.7	910.5 ± 113.6
MCV (fl)	54.3 ± 1.3	54.1 ± 1.3	53.4 ± 0.8	54.7 ± 1
MCH (pg)	18.1 ± 0.49	17.6 ± 1.2	17.8 ± 0.4	18.2 ± 0.4
MCHC (g/dl)	33.4 ± 0.4	32.6 ± 2.1	33.4 ± 0.6	33.4 ± 0.3
Neutrophils (%)	14.6 ± 4.3	13.4 ± 4.2	12.3 ± 3.5	12.6 ± 4.7
Eosinophils (%)	1.1 ± 0.6	1.3 ± 0.4	1.2 ± 0.3	1.2 ± 0.5
Basophils (%)	0.3 ± 0.1	0.3 ± 0.1	0.3 ± 0.1	0.2 ± 0
Lymphocytes (%)	79.4 ± 4.3	81.2 ± 4.7	82.7 ± 4.1	82.2 ± 5.2
Monocytes (%)	2.9 ± 0.7	2.7 ± 1.2	2.1 ± 0.6	2.3 ± 0.9
Reticulocytes (%)	2.9 ± 0.6	3.0 ± 0.7	2.9 ± 0.7	2.9 ± 0.5
PT (sec)	10.3 ± 0.4	10.3 ± 0.4	10.4 ± 0.9	10.4 ± 0.4
aPTT (sec)	34.5 ± 6.7	36.7 ± 2.9	35.3 ± 3.4	34.1 ± 2.7
Female (n = 10/group)				
WBC (10^3^/mm^3^)	8.20 ± 2.27	6.88 ± 1.53	7.28 ± 2.09	7.64 ± 1.69
RBC (10^6^/mm^3^)	7.32 ± 0.12	7.46 ± 0.24	7.59 ± 0.21	7.47 ± 0.29
HGB (g/dl)	13.9 ± 0.2	14 ± 0.2	14.3 ± 0.5	14 ± 0.5
HCT (%)	41.0 ± 0.8	40.9 ± 1.0	42.2 ± 1.6	41.7 ± 1.5
PLT (10^3^/mm^3^)	874.1 ± 69.5	988.8 ± 171.6	943.8 ± 100.1	892 ± 100.1
MCV (fl)	56.1 ± 1.1	54.9 ± 1.2	55.6 ± 1.5	55.8 ± 1.3
MCH (pg)	19.0 ± 0.3	18.7 ± 0.5	18.8 ± 0.5	18.8 ± 0.5
MCHC (g/dl)	33.8 ± 0.4	34.2 ± 0.7	33.9 ± 0.5	33.7 ± 0.5
Neutrophils (%)	12.3 ± 2.8	12.6 ± 2.2	11.0 ± 3.1	11.9 ± 9.4
Eosinophils (%)	1.4 ± 0.6	1.2 ± 0.7	1.1 ± 0.3	1.1 ± 0.5
Basophils (%)	0.3 ± 0.1	0.2 ± 0.1	0.2 ± 0.1	0.2 ± 0.1
Lymphocytes (%)	82.2 ± 2.6	81.8 ± 3.9	83.9 ± 4	82.5 ± 9.4
Monocytes (%)	2.3 ± 0.6	2.6 ± 0.9	2.4 ± 0.6	2.7 ± 0.7
Reticulocytes (%)	2.4 ± 0.4	2.3 ± 0.4	2.4 ± 0.3	2.7 ± 0.4
PT (sec)	9.4 ± 0.3	9.3 ± 0.4	9.2 ± 0.2	9.0 ± 0.3
aPTT (sec)	32.2 ± 2.2	33.2 ± 1.5	32.8 ± 2.1	31.0 ± 3.0

WBC, white blood cells; RBC, red blood cells; Hb, hemoglobin; HCT, hematocrit; PLT, platelet; MCV, mean corpuscular volume; MCH, mean corpuscular hemoglobin; MCHC, mean corpuscular hemoglobin concentration; PT, partial thromboplastin time; and aPTT, activated partial thromboplastin time; BUN, blood urea nitrogen; TC, total cholesterol; TP, total protein; TB, total bilirubin; ALP, alkaline phosphatase; AST, aspartate aminotransferase; ALT, alanine aminotransferase; GGT, γ-glutamyl transferase; and TG, triglycerides.

a *p* < 0.05 by one-way ANOVA followed by post hoc Dunnett’s t-test.

Serum biochemical analysis revealed, when compared to the control, significantly low levels of total cholesterol in the male 5,000 mg/kg group (*p* < 0.05) with a mild dose-dependent pattern of decrease in the lower dose groups (Table 3). The levels of total protein, chlorine, and phosphorus were also observed to be significantly lower in this group, while γ-glutamyl transferase was higher in the male 1,000 mg/kg group. These values were in the published range of normal values (Giknis and Clifford, 2006) or institutional historical data, suggesting them to be non-adverse changes. Unlike the male groups, there was no noticeable change observed in the female groups. In addition, urinalysis (Supplementary Table S1) and ophthalmological examination (data not shown) carried out in the last week of ATWE administration found no abnormal changes.

**TABLE 3:** Serum biochemical parameters of SD rats orally treated with *Acer tegmentosum* Maxim. water extract for 90 days.

	Dose of *Acer tegmentosum* Maxim. water extract (mg/kg)			
	0	1,000	2,000	5,000
Male (n = 10/group)				
BUN (mg/dl)	13.7 ± 1.6	13.8 ± 2.1	13.0 ± 1.5	12.0 ± 2.1
CHOL (mg/dl)	82.0 ± 9.2	79.3 ± 13.9	69.3 ± 10.3	62.2 ± 11.8^a^
TP (g/dl)	6.1 ± 0.3	6.1 ± 0.3	5.9 ± 0.2	5.7 ± 0.2^a^
Albumin (g/dl)	2.3 ± 0.1	2.3 ± 0.1	2.2 ± 0.1	2.2 ± 0.1
TB (mg/dl)	0.04 ± 0.02	0.04 ± 0.02	0.04 ± 0.01	0.04 ± 0.01
ALP (IU/L)	298.1 ± 43.1	304.6 ± 74.6	299.1 ± 64.7	282.7 ± 50.7
AST (IU/L)	96.5 ± 14.5	104.8 ± 23.0	93.4 ± 19.4	95.9 ± 25.9
ALT (IU/L)	32.1 ± 4.7	30.7 ± 4.5	33.3 ± 5.2	28.3 ± 3.5
GGT (IU/L)	0.0 ± 0.0	0.0 ± 0.0	0.3 ± 0.5*	0.1 ± 0.3
Creatinine (mg/dl)	0.48 ± 0.04	0.49 ± 0.04	0.49 ± 0.06	0.49 ± 0.03
TC (mg/dl)	65.4 ± 45.4	57.5 ± 34.3	74.0 ± 39.4	51.8 ± 22.0
Glucose (mg/L)	131.5 ± 12.5	125.2 ± 9.0	134.4 ± 10.4	136.4 ± 18.0
A/G	0.60 ± 0.05	0.60 ± 0.04	0.61 ± 0.03	0.60 ± 0.00
Potassium (mEq/L)	4.6 ± 0.2	4.8 ± 0.2	4.6 ± 0.3	4.5 ± 0.2
Chlorine (mEq/L)	100.1 ± 1.4	99.6 ± 0.7	100.7 ± 1.8	101.9 ± 1.8^a^
Sodium (mEq/L)	141.0 ± 2.0	141.4 ± 1.1	142.4 ± 1.3	142.6 ± 2.2
Calcium (mg/dl)	11.0 ± 0.4	10.9 ± 0.5	10.9 ± 0.2	10.7 ± 0.2
Phosphorous (mg/dl)	7.0 ± 0.4	6.7 ± 0.4	6.8 ± 0.5	6.5 ± 0.3^a^
Female (n = 10/group)				
BUN (mg/dl)	15.6 ± 1.9	13.9 ± 1.8	15.8 ± 3.0	13.8 ± 3.1
CHOL (mg/dl)	77.6 ± 9	78.0 ± 13.8	79.5 ± 16.1	79.1 ± 11.8
TP (g/dl)	5.9 ± 0.4	6.2 ± 0.4	6.2 ± 0.6	6.2 ± 0.4
Albumin (g/dl)	2.6 ± 0.2	2.7 ± 0.3	2.7 ± 0.3	2.7 ± 0.2
TB (mg/dl)	0.04 ± 0.02	0.05 ± 0.03	0.08 ± 0.03	0.07 ± 0.03
ALP (IU/L)	203.9 ± 50.3	146.8 ± 29.6	201.0 ± 76.1	159.2 ± 79.5
AST (IU/L)	96.9 ± 20.9	86.9 ± 20.6	113.6 ± 62.0	96.0 ± 23.6
ALT (IU/L)	26.2 ± 8.0	24.4 ± 5.5	33.2 ± 19.1	29.4 ± 10.6
GGT (IU/L)	0.0 ± 0.0	0.2 ± 0.4	0.2 ± 0.4	0.2 ± 0.4
Creatinine (mg/dl)	0.55 ± 0.08	0.50 ± 0.03	0.51 ± 0.04	0.48 ± 0.05
TC (mg/dl)	30.8 ± 21.6	43.8 ± 22.3	41.8 ± 19.7	52.2 ± 33.0
Glucose (mg/L)	127.4 ± 13.8	139.1 ± 16.2	124.4 ± 15.1	134.7 ± 9.5
A/G	0.77 ± 0.07	0.77 ± 0.08	0.76 ± 0.05	0.77 ± 0.05
Potassium (mEq/L)	4.3 ± 0.4	4.4 ± 0.3	4.2 ± 0.3	4.2 ± 0.3
Chlorine (mEq/L)	100.6 ± 1.8	100.9 ± 0.9	99.2 ± 1.5	101.1 ± 1.9
Sodium (mEq/L)	139.3 ± 2.2	139.2 ± 1.8	139.6 ± 1.6	140.9 ± 2.3
Calcium (mg/dl)	10.6 ± 0.4	11.0 ± 0.3	10.8 ± 0.6	11.0 ± 0.5
Phosphorous (mg/dl)	6.1 ± 0.8	5.8 ± 0.9	6.2 ± 0.5	6.0 ± 0.7

BUN, blood urea nitrogen; TC, total cholesterol; TP, total protein; TB, total bilirubin; ALP, alkaline phosphatase; AST, aspartate aminotransferase; ALT, alanine aminotransferase; GGT, γ-glutamyl transferase; and TG, triglycerides.

a *p* < 0.05 by one-way ANOVA followed by post hoc Dunnett’s t-test.

All surviving animals were sacrificed after finishing the 90-day administration schedule and subjected to necropsy. When gross examination was performed, sporadic cases of minor lesions such as discoloration and redness in several organs were identified across the control and treatment groups, but no lesions were considered to be associated with ATWE administration (Supplementary Table S2). The measurement of wet organ weight (Table 4) revealed that the absolute and relative weights of the spleen were significantly reduced in the male 5,000 mg/kg group (*p* < 0.05 in all). In the female groups, the absolute weight of the left kidney in 2,000 and 5,000 mg/kg groups was significantly higher than that in the control group (*p* < 0.05), while a higher relative weight was observed in the liver of the 5,000 mg/kg group (*p* < 0.05) and in both sides of the kidneys in 2,000 and 5,000 mg/kg groups (*p* < 0.05 in all). For further investigation of microscopic lesions, the histopathological analysis was performed on the control and highest dose groups. Despite the changes in organ weight, there were no test substance–related pathologies observed in any organs besides spontaneous or incidental lesions (Supplementary Table S3). These findings indicate that ATWE did not cause toxicity in the repeated-dose study, establishing that the NOAEL of ATWE in SD rats is >5,000 mg/kg BW.

**TABLE 4:** Absolute and relative weights of major organs from SD rats orally treated with *Acer tegmentosum* Maxim. water extract for 13 weeks.

		Dose of *Acer tegmentosum* Maxim. water extract (mg/kg)						Dose of *Acer tegmentosum* Maxim. water extract (mg/kg)			
		0	1,000	2000	5,000			0	1,000	2000	5,000
Male (n = 10/group)						Female (n = 10/group)					
Liver	(g)	17.39 ± 3.22	17.86 ± 2.27	16.53 ± 1.95	15.23 ± 2.40	Liver	(g)	8.65 ± 0.90	8.97 ± 0.69	9.26 ± 0.66	9.25 ± 1.18
	(g%)	2.88 ± 0.26	2.94 ± 0.22	2.83 ± 0.18	2.66 ± 0.35		(g%)	2.68 ± 0.18	2.78 ± 0.11	2.87 ± 0.30	2.95 ± 0.21^a^
Spleen	(g)	1.03 ± 0.18	1.08 ± 0.22	0.92 ± 0.10	0.85 ± 0.13^a^	Spleen	(g)	0.63 ± 0.10	0.58 ± 0.06	0.63 ± 0.07	0.63 ± 0.05
	(g%)	0.17 ± 0.03	0.18 ± 0.02	0.16 ± 0.01	0.15 ± 0.02^a^		(g%)	0.19 ± 0.03	0.18 ± 0.02	0.20 ± 0.03	0.20 ± 0.01
Kidney (R)	(g)	1.77 ± 0.21	1.68 ± 0.15	1.61 ± 0.15	1.65 ± 0.16	Kidney (R)	(g)	0.95 ± 0.08	0.99 ± 0.08	1.05 ± 0.08	1.00 ± 0.09
	(g%)	0.30 ± 0.02	0.28 ± 0.02	0.28 ± 0.02	0.29 ± 0.03		(g%)	0.29 ± 0.02	0.31 ± 0.03	0.32 ± 0.02^a^	0.32 ± 0.01^a^
Kidney (L)	(g)	1.70 ± 0.16	1.67 ± 0.14	1.63 ± 0.16	1.66 ± 0.16	Kidney (L)	(g)	0.92 ± 0.05	0.98 ± 0.09	1.04 ± 0.08^a^	1.02 ± 0.08^a^
	(g%)	0.28 ± 0.02	0.28 ± 0.02	0.28 ± 0.03	0.29 ± 0.03		(g%)	0.29 ± 0.02	0.31 ± 0.03	0.32 ± 0.02^a^	0.33 ± 0.01^a^
Adrenal gl. (R)	(g)	0.027 ± 0.003	0.027 ± 0.003	0.028 ± 0.004	0.028 ± 0.005	Adrenal gl. (R)	(g)	0.038 ± 0.006	0.033 ± 0.004	0.036 ± 0.005	0.035 ± 0.006
	(g%)	0.005 ± 0.000	0.004 ± 0.001	0.005 ± 0.000	0.005 ± 0.001		(g%)	0.012 ± 0.002	0.010 ± 0.001	0.011 ± 0.002	0.012 ± 0.002
Adrenal gl. (L)	(g)	0.028 ± 0.004	0.029 ± 0.003	0.029 ± 0.006	0.029 ± 0.006	Adrenal gl. (L)	(g)	0.039 ± 0.006	0.034 ± 0.004	0.037 ± 0.006	0.038 ± 0.004
	(g%)	0.005 ± 0.000	0.005 ± 0.001	0.005 ± 0.001	0.005 ± 0.001		(g%)	0.012 ± 0.002	0.010 ± 0.001	0.011 ± 0.002	0.011 ± 0.002
Testis (R)	(g)	1.78 ± 0.19	1.73 ± 0.15	1.74 ± 0.18	1.76 ± 0.11	Ovary (R)	(g)	0.05 ± 0.01	0.04 ± 0.01	0.04 ± 0.01	0.04 ± 0.01
	(g%)	0.30 ± 0.03	0.29 ± 0.03	0.30 ± 0.04	0.31 ± 0.03		(g%)	0.01 ± 0.00	0.01 ± 0.00	0.01 ± 0.00	0.01 ± 0.00
Testis (L)	(g)	1.79 ± 0.19	1.72 ± 0.14	1.73 ± 0.19	1.75 ± 0.11	Ovary (L)	(g)	0.05 ± 0.01	0.04 ± 0.00	0.05 ± 0.01	0.04 ± 0.01
	(g%)	0.30 ± 0.03	0.28 ± 0.03	0.30 ± 0.04	0.31 ± 0.03		(g%)	0.01 ± 0.00	0.01 ± 0.00	0.01 ± 0.00	0.01 ± 0.00
Thymus	(g)	0.30 ± 0.09	0.27 ± 0.08	0.24 ± 0.06	0.26 ± 0.09	Thymus	(g)	0.28 ± 0.09	0.24 ± 0.05	0.27 ± 0.05	0.26 ± 0.05
	(g%)	0.05 ± 0.01	0.04 ± 0.01	0.04 ± 0.01	0.05 ± 0.02		(g%)	0.09 ± 0.03	0.08 ± 0.02	0.08 ± 0.01	0.08 ± 0.02
Heart	(g)	1.68 ± 0.19	1.69 ± 0.21	1.60 ± 0.16	1.60 ± 0.16	Heart	(g)	1.00 ± 0.10	1.03 ± 0.09	1.08 ± 0.05	1.01 ± 0.10
	(g%)	0.28 ± 0.01	0.28 ± 0.02	0.27 ± 0.02	0.28 ± 0.02		(g%)	0.31 ± 0.02	0.32 ± 0.02	0.33 ± 0.02	0.32 ± 0.03
Lung	(g)	1.68 ± 0.11	1.73 ± 0.23	1.62 ± 0.12	1.70 ± 0.17	Lung	(g)	1.31 ± 0.14	1.28 ± 0.18	1.36 ± 0.14	1.28 ± 0.13
	(g%)	0.28 ± 0.02	0.28 ± 0.02	0.28 ± 0.03	0.30 ± 0.02		(g%)	0.41 ± 0.03	0.40 ± 0.05	0.42 ± 0.05	0.41 ± 0.04
Brain	(g)	2.20 ± 0.08	2.21 ± 0.10	2.17 ± 0.08	2.17 ± 0.09	Brain	(g)	2.01 ± 0.07	2.00 ± 0.09	2.03 ± 0.10	2.00 ± 0.06
	(g%)	0.37 ± 0.04	0.37 ± 0.03	0.38 ± 0.04	0.38 ± 0.03		(g%)	0.63 ± 0.07	0.62 ± 0.06	0.63 ± 0.07	0.64 ± 0.04
Pituitary gl	(g)	0.0153 ± 0.0021	0.0151 ± 0.0018	0.0147 ± 0.0013	0.0147 ± 0.0015	Pituitary gl	(g)	0.0188 ± 0.0027	0.0186 ± 0.0021	0.0201 ± 0.0047	0.0178 ± 0.0020
	(g%)	0.0026 ± 0.0003	0.0025 ± 0.0003	0.0025 ± 0.0002	0.0026 ± 0.0003		(g%)	0.0059 ± 0.0010	0.0058 ± 0.0010	0.0062 ± 0.0015	0.0057 ± 0.0005

a *p* < 0.05 by one-way ANOVA followed by post hoc Dunnett’s t-test.

### Acute Oral Toxicity Study in Beagle Dogs

**FIGURE 4:**
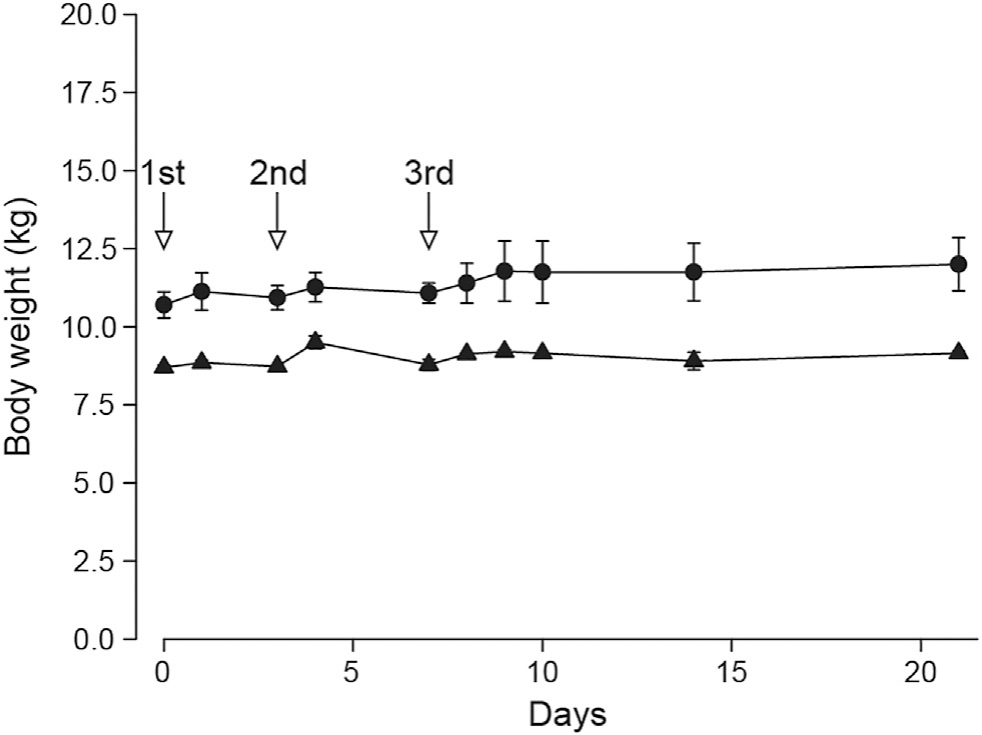
Normal body weight of beagle dogs treated with *Acer tegmentosum* Maxim. water extract in the acute toxicity study. Male and female animals (n = 2/gender) were sequentially administered an increasing dose of *Acer tegmentosum* Maxim. water extract (ATWE; 1st administration, 1,000 mg/kg BW; 2nd, 2,000 mg/kg BW; and 3rd, 5,000 mg/kg BW) with an interval of 3–4 days after the 1st and 2nd administration and 14 days after the 3rd administration for monitoring clinical signs and body weight. All animals showed a normal range of body weight during and after treatment of ATWE.

The general toxicity study in SD rats showed ATWE was safe in the tested dose range. In order to test the toxicity of ATWE in non-rodent animals, we performed an acute oral toxicity study in beagle dogs. Male and female animals (n = 2/gender) received single oral administration of increasing ATWE doses, namely, 1,000, 2,000, and 5,000 mg/kg BW with an interval of 3–4 days for clinical observation after the 1st and the 2nd administration and 2 weeks after the last administration. During the whole period of the study, no animals showed mortality (data not shown) or noticeable alteration in the body weight (Figure 4). Clinically, all animals were observed to be normal after 1,000 and 2000 mg/kg BW of ATWE. Although intermittent vomiting was noted in all animals 0.5–2 h after administration of 5,000 mg/kg, it was temporary in its nature without accompaniment of any other clinical signs or behavioral abnormality, suggesting that vomit was caused by physical stress associated with the highly concentrated test substance to the digestive system rather than its toxicity. When examined for necropsy, no pathological lesions or abnormality likely linked to the test substance was observed in any animals. These findings indicate that LD50 of ATWE in beagle dogs is >5,000 mg/kg BW.

### Bacterial Reverse Mutation Test

**FIGURE 5:**
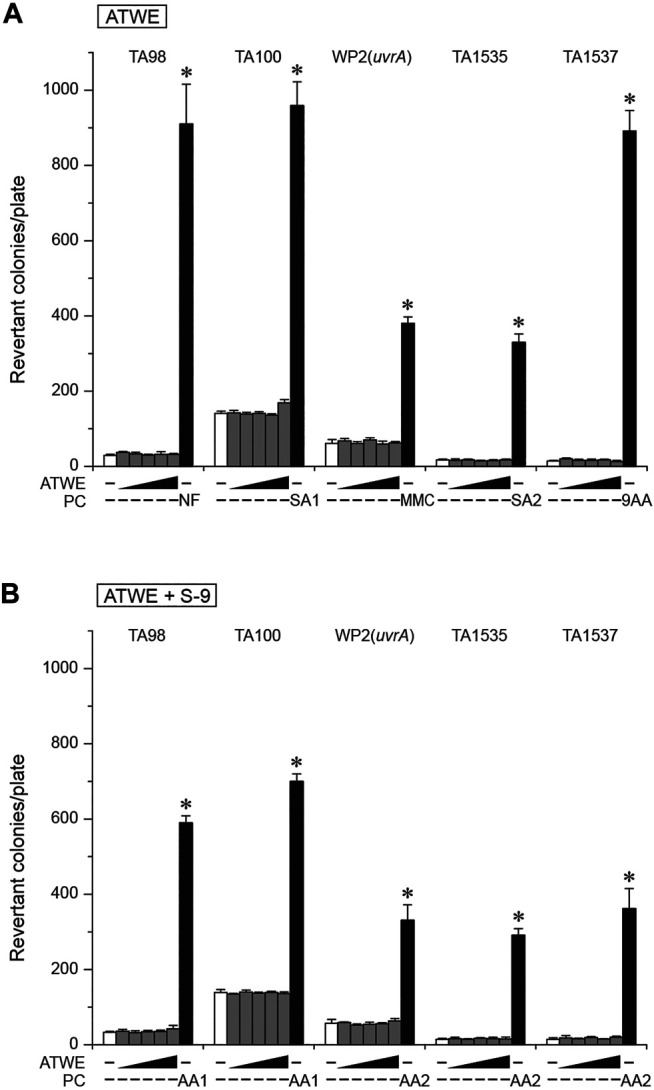
Absence of mutagenicity in the bacterial reverse mutation test of *Acer tegmentosum* Maxim. water extract. *Acer tegmentosum* Maxim. water extract (ATWE) was tested on five bacterial tester strains to evaluate mutagenic potentials. When treated with one of 0, 312.5, 625, 1,250, 2,500, and 5,000 µg/plate ATWE, **(A)** none of the tester strains showed an increase of revertant colonies compared to the vehicle controls except for a mild elevation in TA100 treated with the highest dose, and **(B)** metabolic activation of ATWE with S-9 mixture resulted in comparable levels of revertant colonies to the vehicle control in each tester strain, while positive controls significantly increased the number. White bars; vehicle control, dark gray bars; ATWE, black bars; positive controls, NF; 10.0 µg/plate 2-nitrofluorene, SA1; 5.0 µg/plate of sodium azide, SA2; 0.5 µg/plate of sodium azide, 9AA; 80.0 µg/plate of 9-aminoacridine, AA1; 2.0 µg/plate of 2-aminoanthracene and AA2; 5.0 µg/plate of 2-aminoanthracene. *; *p* < 0.05 by one-way ANOVA followed by *post hoc* Dunnett’s t-test.

A previous study reported that an extract of *A. tegmentosum* significantly increased revertant colonies (Hwang et al., 2013). Despite its potential significance, there have been no other attempts made to confirm the results. Therefore, we performed a bacterial reverse mutation test in order to assess the mutagenicity of our ATWE. All the tested doses of ATWE were not cytotoxic in *S. typhimurium* TA100 tester strain (Supplementary Figure S2). When tested in 5 tester strains (Figure 5), ATWE increased the number of revertant colonies only in TA100 to a mild degree of 1.2 folds at the highest dose. Excluding this, the number of colonies in all tester strains treated with ATWE was similar to the vehicle control, regardless of metabolic activation with S-9 mixture. In sharp contrast, the respective positive controls remarkably increased the number of colonies, confirming validity of the performed tests. Collectively, our results showed that ATWE did not increase revertant colonies in the bacterial reverse mutation test, suggesting that it is not mutagenic.

### *In vitro* Chromosome Aberration Test

**FIGURE 6:**
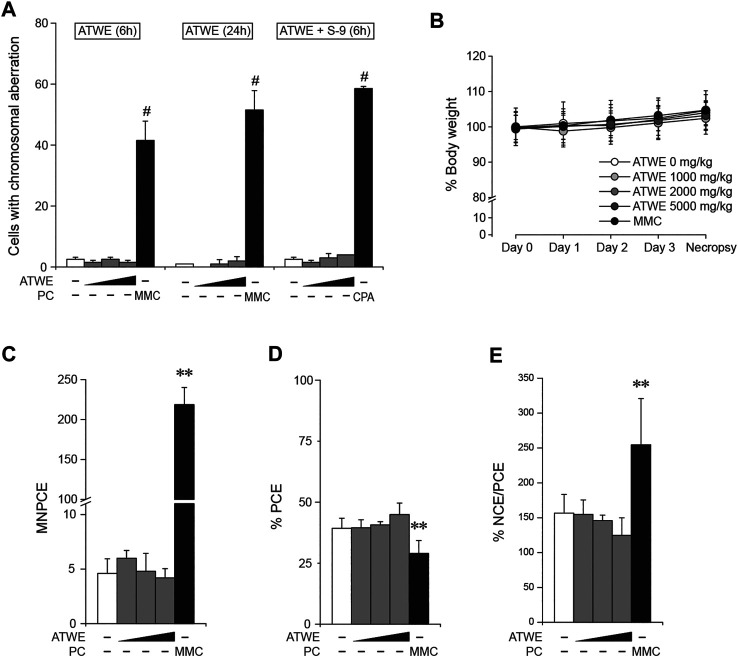
*Acer tegmentosum* Maxim. water extract was not clastogenic. Chinese hamster lung (CHL) cells were treated with 0, 125, 250, and 500 µg/ml of *Acer tegmentosum* Maxim. water extract (ATWE) for the 6- and 24-h treatment groups, and 0, 625, 1,250, and 2,500 µg/ml for the 6-h treatment with metabolic activation using S-9 mixture for *in vitro* chromosome aberration test. **(A)** ATWE showed similar numbers of cells containing aberrant chromosomes to the vehicle control, while positive control groups showed significant increase. For *in vivo* micronucleus test, 8-week-old male ICR mice (n = 5/group) were orally administered either 1,000, 2,000, or 5,000 mg/kg BW of ATWE for 4 days. **(B)** During the administration, body weight of animals was comparable among the groups regardless of the treated doses. **(C)** ATWE did not increase the number of micronucleus-containing polychromatic erythrocytes (MNPCEs), **(D)** percentage of PCE **(E)** nor percentage ratio of normochromatic erythrocytes (NCEs) and PCE, while the positive control (PC) induced significant changes compared to the vehicle control. White symbols, vehicle control; gray symbols, ATWE; black symbols, positive control; MMC, mitomycin C; CPA, cyclophosphamide. #; *p* < 0.05 when compared to the control by Fisher’s exact test and **; *p* < 0.01 by the Kruskal–Wallis test followed by *post hoc* Tukey’s HSD multiple comparison test.

Next, we tested whether ATWE was clastogenic *in vitro* using a chromosome aberration test employing CHL cells. ATWE induced cytotoxicity in the cells at the high range of doses, and the toxicity was more prominent when treated without S-9 mixture (data not shown). Accordingly, CHL cells were treated with the highest dose of either 500 µg/ml for 6 and 24 h or 2,500 µg/ml plus S-9 mixture for 6 h (Figure 6A). Unlike the significant increase by mitomycin C or cyclophosphamide, the number of cells containing aberrant chromosomes remained unchanged in any ATWE doses and treatment conditions, suggesting that ATWE was not clastogenic in CHL cells.

### *In vivo* Micronucleus Test

The previous study showed that ATWE was not genotoxic *in vitro*. To confirm the findings in an *in vivo* system, we carried out a micronucleus test using ICR mice. When ATWE was initially tested to determine the maximum dose and appropriate sampling time after dosing, 5,000 mg/kg BW for 4 days and 24 h after the administration were selected for ATWE (Supplementary Figure S3), and therefore, we carried out the main test under these conditions (Figure 6B–E). During the administration period, all animals showed a similar body weight without manifesting mortality or abnormal behavior. Contrary to the effect of mitomycin C, ATWE did not increase the number of micronucleus-containing polychromatic erythrocytes (MNPCEs). In addition, the percentage of PCE, and normochromatic erythrocytes (NCEs)/PCE in ATWE-treated groups remained similar to those in the vehicle control. These findings indicate that ATWE did not induce micronucleus nor involve in the suppression of the bone marrow cells, suggesting lack of *in vivo* clastogenicity in ATWE.

## Discussion

*A. tegmentosum* has been a traditional ingredient mainly in Asian folk medicine with several therapeutic applications. A growing body of knowledge on the therapeutically active components and their beneficial effects has drawn increasing attention to the supplements and functional foods containing *A. tegmentosum*–derived products. Despite the nature of frequent and long-term use as an herbal supplement, safety information on *A. tegmentosum* remained largely lacking. In this study, we therefore systematically evaluated the safety of *A. tegmentosum* by performing acute and subchronic repeated oral toxicity studies of ATWE in SD rats and an acute study in beagle dogs with concurrent reevaluation of genotoxicity. To our best knowledge, this is the first report on the NOAEL of *A. tegmentosum* with the lack of a target organ from the repeated-dose oral toxicity test in rodents, its safety in a non-rodent species, as well as lack of mutagenic potential. Collectively, our study provides the essential information for the safe human use of *A. tegmentosum* by covering general toxicity assessment in rodent and non-rodent species as well as genotoxicity.

In the acute toxicity study in SD rats, 5,000 mg/kg BW of ATWE did not cause any toxic signs in all the parameters measured, establishing its LD50 to be >5,000 mg/kg BW. These findings were closely in line with the results of the oral toxicity test performed on an *A. tegmentosum* extract (Hwang et al., 2013), which reported its LD50 to be >2000 mg/kg BW. Subsequently, in the repeated oral study, administration of up to 5,000 mg/kg BW ATWE for 90 days did not alter body weight or affect other physiological readouts, including clinical signs, and food and water intake. Moreover, analyses performed on necropsy revealed no ATWE-associated abnormality in hematology, serum biochemistry, and histopathology excluding spontaneous or incidental changes, determining for the first time that the NOAEL of ATWE in SD rats is >5,000 mg/kg BW without a recognizable target organ. These results in turn indicated that salidroside contained in the highest dose of ATWE (371.5 mg/kg based on 74.3 ± 0.8 mg/g) did not cause systemic toxicity. Recently, Lu et al. showed that 276 mg/kg of salidroside included in an *Osmanthus fragrans* flower extract did not cause any noticeable toxicity in a 90-day repeated toxicity study (Lu et al., 2016). In the current lack of toxicity information on pure salidroside, our study, suggested the safety use of salidroside included in herbal extracts despite the difference in the test substance and coexisting components, and significantly broadened its potential safe dose range. However, interpretation and application of these results may require caution as the influence of other compounds on the biological activity of salidroside has not been assessed. Lastly, the acute toxicity study we performed in beagle dogs showed that 5,000 mg/kg BW of ATWE was safe, determining that its initial LD50 in the non-rodent species is >5,000 mg/kg BW. Taken together, these results suggested that the tested dose range of ATWE is nontoxic in the rodent and non-rodent animal models used in this study and significantly increased the spectrum of toxicity information on *A. tegmentosum* from the previous reports.

When we assessed mutagenicity of ATWE in this study, the number of revertant colonies did not increase in any tester strains, except for the mild elevation detected in TA100 at the highest dose, contradicting the previous findings of a significant increase in TA98, regardless of S-9 co-treatment with a tendency of dose-dependent increase in TA100, TA1537, and WP2 (*uvrA*) tester strains (Hwang et al., 2013). Currently, it is not clear what induced the discrepancy between two studies, but possible causes could be 1) the use of raw materials from different sources or different parts of the plant or 2) difference in the preparation of test substances. It is of note that our study also showed an increase observed in TA100 although statistically insignificant. Therefore, it is conceivable that our test substance may have less amount of the compound(s) which caused higher increase of revertant colonies in the previous study. Notably, a study reported that composition of phenolic compounds in *A. tegmentosum* was markedly different among its parts (Lee et al., 2017a). Moreover, several genotoxicity studies showed lack thereof in a handful of the components listed in Table 1, such as catechin (Ogura et al., 2008), salidroside (Zhu et al., 2010), quercetin (Harwood et al., 2007), myricitrin, and myricetin (Hobbs et al., 2015), excluding their causative involvement in the previously observed mutagenicity. However, many still remained to be tested. To clarify the issue, further investigation may be warranted to compare the components and their levels among *A. tegmentosum* from different sources and its parts with acquiring information on genotoxicity of such components.

In the present study, we assessed systemic and genetic toxicity of ATWE and demonstrated that ATWE did not cause acute toxicity in SD rats and beagle dogs and subchronic toxicity in SD rats at the doses up to 5,000 mg/kg BW. Our results showed that there was no general or organ-specific toxicity caused by ATWE with no target organ identified. In addition, we showed that ATWE was not genotoxic. Collectively, our study provided the first comprehensive information on the nonclinical safety of ATWE, providing the urgently required toxicity information and an informative guideline on the safe use of *A. tegmentosum* in humans.

## Conclusion

We systemically evaluated the acute and repeated oral toxicity of *A. tegmentosum* Maxim. water extract in this study and found that LD50 in SD rats and beagle dogs is >5,000 mg/kg BW, and the NOAEL in SD rats is >5,000 mg/kg BW. In addition, our results from mutagenicity and clastogenicity tests demonstrated that ATWE is not genotoxic. Our findings provide the initial and comprehensive information for the safe use of *A. tegmentosum* in humans. Of note, these results were based on the test substance derived from exclusive use of twigs. Due to the potential difference in toxicity, therefore, caution should be exercised in application of the present results to other products containing different parts of *A. tegmentosum* until confirmation of safety is made through further studies.

## Data Availability

The original contributions presented in the study are included in the article/Supplementary Material, and further inquiries can be directed to the corresponding author.
