# Supplementary figures and images for: Safety Assessment of Acer tegmentosum Maxim. Water Extract: General Toxicity Studies in Sprague–Dawley Rats and Beagle Dogs With Re-evaluation of Genotoxic Potentials
Source: Front Pharmacol. 2021 Aug 31;12:687261. doi: 10.3389/fphar.2021.687261 (PMC8438563; doi:10.3389/fphar.2021.687261)

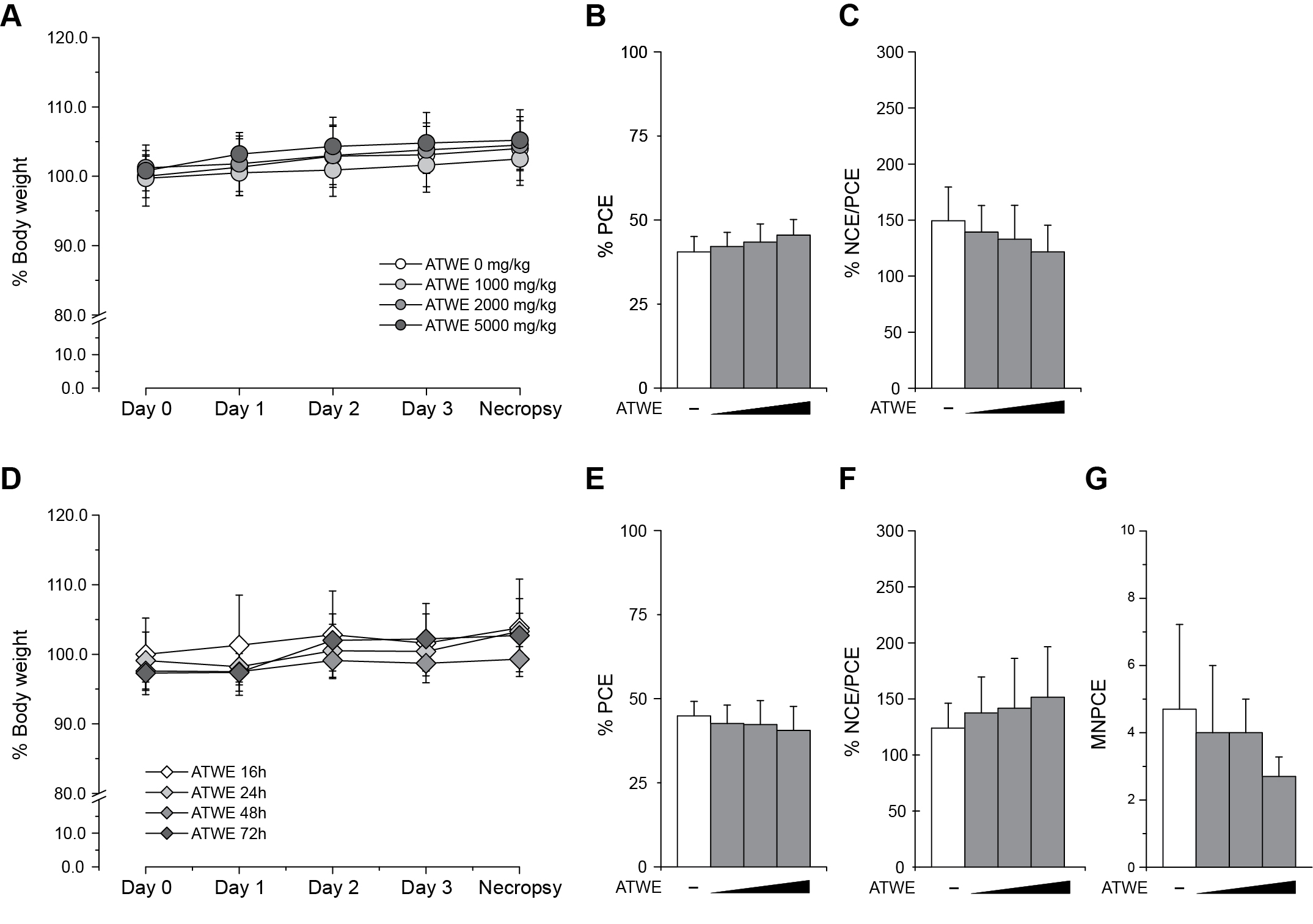

Supplement: Supplementary file 1 [file Image3.JPEG]

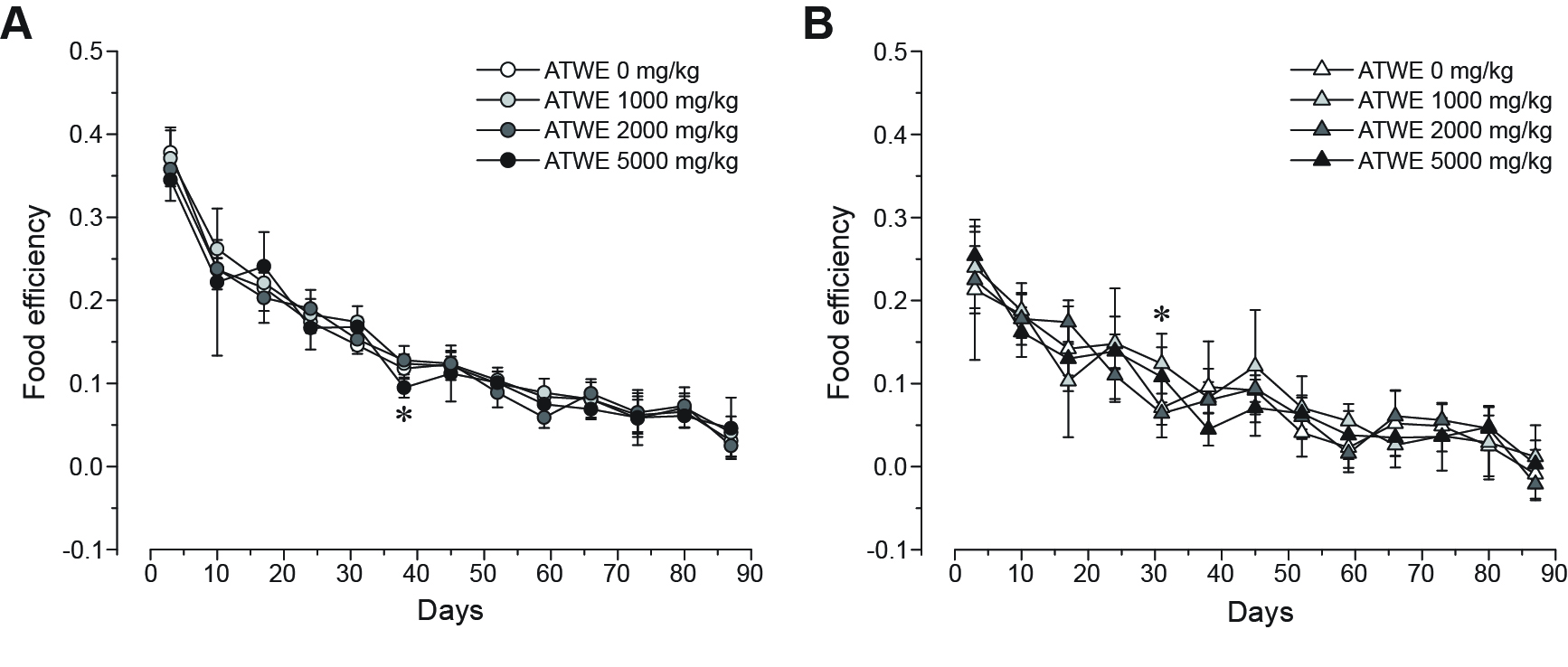

Supplement: Supplementary file 3 [file Image1.JPEG]

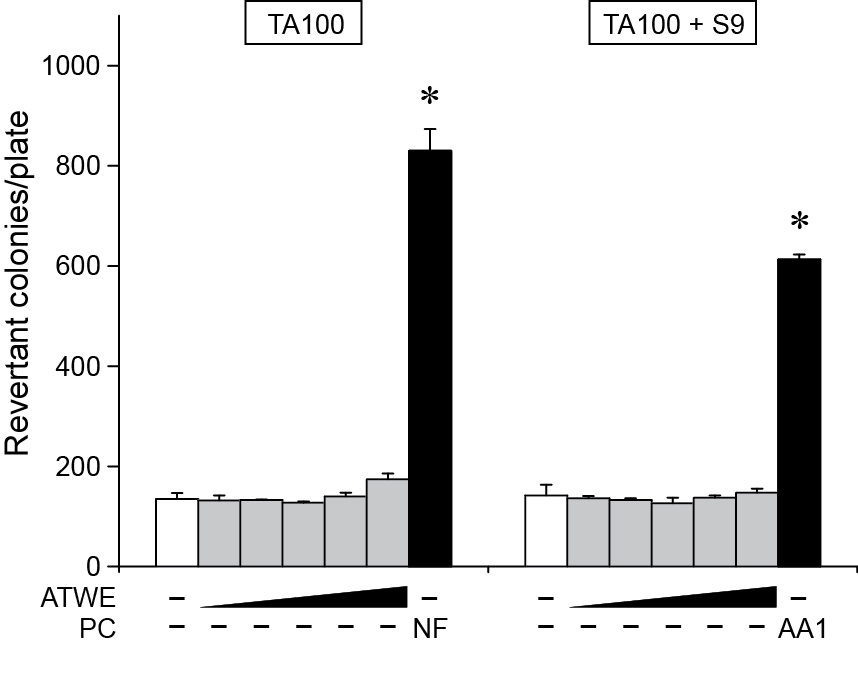

Supplement: Supplementary file 4 [file Image2.JPEG]
