# Supplementary material for: Safety Assessment of Acer tegmentosum Maxim. Water Extract: General Toxicity Studies in Sprague–Dawley Rats and Beagle Dogs With Re-evaluation of Genotoxic Potentials
Source: Front Pharmacol. 2021 Aug 31;12:687261. doi: 10.3389/fphar.2021.687261 (PMC8438563; doi:10.3389/fphar.2021.687261)
Supplement: Supplementary file 2 [file Table1.docx]

| Supplementary Table 1. Urinalysis of SD rats orally treated with *Acer tegmentosum* water extract for 90 days | | | | | | | | | | |
| --- | --- | --- | --- | --- | --- | --- | --- | --- | --- | --- |
| Parameter | Value | Dose of *Acer tegmentosum* water extract (mg/kg) | | | | | | | | |
|  |  |  | Male (n=5/group) | | |  |  | Female (n=5/group) | |  |
|  |  | 0 | 1000 | 2000 | 5000 |  | 0 | 1000 | 2000 | 5000 |
| Specific gravity |  | 1.038 ±0.008 | 1.034 ±0.011 | 1.027 ±0.010 | 1.027 ±0.016 |  | 1.027 ±0.007 | 1.018 ±0.003 | 1.035 ±0.015 | 1.042 ±0.011 |
| pH | 6.0 | 0/5 | 0/5 | 0/5 | 0/5 |  | 0/5 | 0/5 | 1/5 | 0/5 |
|  | 7.0 | 0/5 | 0/5 | 1/5 | 0/5 |  | 2/5 | 1/5 | 0/5 | 0/5 |
|  | 7.5 | 0/5 | 1/5 | 0/5 | 0/5 |  | 1/5 | 0/5 | 1/5 | 0/5 |
|  | 8.0 | 3/5 | 0/5 | 1/5 | 3/5 |  | 1/5 | 2/5 | 1/5 | 2/5 |
|  | 8.5 | 2/5 | 4/5 | 3/5 | 2/5 |  | 1/5 | 2/5 | 2/5 | 2/3 |
| Leukocytes | negative | 1/5 | 2/5 | 3/5 | 2/5 |  | 5/5 | 5/5 | 5/5 | 3/5 |
| (cells/µL) | moderate | 0/5 | 1/5 | 0/5 | 0/5 |  | 0/5 | 0/5 | 0/5 | 0/5 |
|  | trace | 3/5 | 1/5 | 2/5 | 3/5 |  | 0/5 | 0/5 | 0/5 | 1/5 |
|  | small | 1/5 | 1/5 | 0/5 | 0/5 |  | 0/5 | 0/5 | 0/5 | 1/5 |
| Nitrite | - | 5/5 | 5/5 | 5/5 | 5/5 |  | 1/5 | 2/5 | 0/5 | 4/5 |
|  | + | 0/5 | 0/5 | 0/5 | 0/5 |  | 4/5 | 3/5 | 5/5 | 1/5 |
| Protein | negative | 0/5 | 0/5 | 0/5 | 2/5 |  | 2/5 | 5/5 | 2/5 | 2/5 |
| (mg/dl) | trace | 0/5 | 1/5 | 4/5 | 0/5 |  | 2/5 | 0/5 | 1/5 | 0/5 |
|  | 30 | 4/5 | 3/5 | 1/5 | 2/5 |  | 1/5 | 0/5 | 2/5 | 1/5 |
|  | 100 | 1/5 | 1/5 | 0/5 | 1/5 |  | 0/5 | 0/5 | 0/5 | 2/5 |
| Glucose (mg/dl) | negative | 5/5 | 5/5 | 5/5 | 5/5 |  | 5/5 | 5/5 | 5/5 | 5/5 |
| Ketone | negative | 0/5 | 0/5 | 1/5 | 2/5 |  | 5/5 | 5/5 | 4/5 | 1/5 |
| (mg/dl) | trace | 0/5 | 1/5 | 1/5 | 1/5 |  | 0/0 | 0/0 | 1/5 | 3/5 |
|  | 15 | 5/5 | 4/5 | 3/5 | 2/5 |  | 0/0 | 0/0 | 0/0 | 1/5 |
| Urobilinogen | 0.2 | 5/5 | 4/5 | 5/5 | 4/5 |  | 5/5 | 5/5 | 4/5 | 2/5 |
| (E.U./dl) | 1 | 0/5 | 1/5 | 0/5 | 1/5 |  | 0/5 | 0/5 | 1/5 | 3/5 |
| Bilirubin | negative | 5/5 | 4/5 | 5/5 | 5/5 |  | 5/5 | 5/5 | 5/5 | 4/5 |
|  | small | 0/5 | 1/5 | 0/5 | 0/5 |  | 0/5 | 0/5 | 0/5 | 1/5 |
| Blood | negative | 4/5 | 4/5 | 2/4 | 4/5 |  | 5/5 | 5/5 | 5/5 | 5/5 |
|  | moderate | 1/5 | 1/5 | 0/5 | 0/5 |  | 0/5 | 0/5 | 0/5 | 0/5 |
|  | small | 0/5 | 0/5 | 1/5 | 0/5 |  | 0/5 | 0/5 | 0/5 | 0/5 |
|  | trace-intact | 0/5 | 0/5 | 2/5 | 1/5 |  | 0/5 | 0/5 | 0/5 | 0/5 |
