# Supplementary material for: Safety Assessment of Acer tegmentosum Maxim. Water Extract: General Toxicity Studies in Sprague–Dawley Rats and Beagle Dogs With Re-evaluation of Genotoxic Potentials
Source: Front Pharmacol. 2021 Aug 31;12:687261. doi: 10.3389/fphar.2021.687261 (PMC8438563; doi:10.3389/fphar.2021.687261)
Supplement: Supplementary file 5 [file Table2.docx]

| Supplementary table 2. Gross findings in major organs from SD rats orally treated with *Acer tegmentosum* water extract for 90 days | | | | | | | | | | |
| --- | --- | --- | --- | --- | --- | --- | --- | --- | --- | --- |
| Organ | Findings | Dose of *Acer tegmentosum* extract (mg/kg) | | | | | | | | |
|  |  | Male (n=10/group) | | | |  | Female (n=10/group) | | | |
|  |  | 0 | 1000 | 2000 | 5000 |  | 0 | 1000 | 2000 | 5000 |
| Liver | Normal | 10/10 | 9/10 | 10/10 | 7/10 |  | 10/10 | 10/10 | 10/10 | 9/10 |
|  | Spot | 0/10 | 1/10 | 0/10 | 0/10 |  | 0/10 | 0/10 | 0/10 | 0/10 |
|  | Discoloration | 0/10 | 0/10 | 0/10 | 2/10 |  | 0/10 | 0/10 | 0/10 | 0/10 |
|  | Thickened | 0/10 | 0/10 | 0/10 | 1/10 |  | 0/10 | 0/10 | 0/10 | 0/10 |
|  | Nodule | 0/10 | 0/10 | 0/10 | 0/10 |  | 0/10 | 0/10 | 0/10 | 1/10 |
| Lung | Normal | 10/10 | 9/10 | 8/10 | 10/10 |  | 9/10 | 10/10 | 9/10 | 9/10 |
|  | Spot | 0/10 | 1/10 | 1/10 | 0/10 |  | 0/10 | 0/10 | 0/10 | 1/10 |
|  | Discoloration | 0/10 | 0/10 | 1/10 | 0/10 |  | 0/10 | 0/10 | 0/10 | 0/10 |
|  | Redness | 0/10 | 0/10 | 0/10 | 0/10 |  | 1/10 | 0/10 | 1/10 | 0/10 |
| Thymus | Normal | 10/10 | 10/10 | 10/10 | 9/10 |  | 10/10 | 10/10 | 10/10 | 10/10 |
|  | Redness | 0/10 | 0/10 | 0/10 | 1/10 |  | 0/10 | 0/10 | 0/10 | 0/10 |
| Rectum | Normal | 10/10 | 10/10 | 9/10 | 10/10 |  | 10/10 | 10/10 | 10/10 | 9/10 |
|  | Nodule | 0/10 | 0/10 | 1/10 | 0/10 |  | 0/10 | 0/10 | 0/10 | 1/10 |
| Preputial gland | Normal | 10/10 | 10/10 | 10/10 | 9/10 |  | - | - | - | - |
|  | Atrophy | 0/10 | 0/10 | 0/10 | 1/10 |  | - | - | - | - |
| Clitoral gland | Normal | - | - | - | - |  | 9/10 | 10/10 | 10/10 | 8/10 |
|  | Redness | - | - | - | - |  | 1/10 | 0/10 | 0/10 | 0/10 |
|  | Mass | - | - | - | - |  | 0/10 | 0/10 | 0/10 | 1/10 |
|  | Discoloration | - | - | - | - |  | 0/10 | 0/10 | 0/10 | 1/10 |
| Abdomen | Normal | 10/10 | 10/10 | 10/10 | 10/10 |  | 9/10 | 9/10 | 10/10 | 10/10 |
|  | Mass | 0/10 | 0/10 | 0/10 | 0/10 |  | 1/10 | 1/10 | 0/10 | 0/10 |
