# Supplementary material for: Safety Assessment of Acer tegmentosum Maxim. Water Extract: General Toxicity Studies in Sprague–Dawley Rats and Beagle Dogs With Re-evaluation of Genotoxic Potentials
Source: Front Pharmacol. 2021 Aug 31;12:687261. doi: 10.3389/fphar.2021.687261 (PMC8438563; doi:10.3389/fphar.2021.687261)
Supplement: Supplementary file 6 [file Table3.docx]

| Supplementary Table 3. Histopathological findings in major organs from SD rats orally treated with *Acer tegmentosum* water extract for 90 days | | | | | | | |
| --- | --- | --- | --- | --- | --- | --- | --- |
| Organ | Findings |  | Dose of *Acer tegmentosum* water extract (mg/kg) | | | | |
|  |  |  | Male  (n=10/group) | |  | Female (n=10/group) | |
|  |  |  | 0 | 5000 |  | 0 | 5000 |
| *Nervous system* |  |  |  |  |  |  |  |
| Brain | Normal |  | 10/10 | 10/10 |  | 10/10 | 10/10 |
| Sciatic nerve | Normal |  | 10/10 | 10/10 |  | 10/10 | 10/10 |
| *Ocular system* |  |  |  |  |  |  |  |
| Eyes | Normal |  | 10/10 | 10/10 |  | 10/10 | 10/10 |
| Harderian glands | Normal |  | 9/10 | 10/10 |  | 10/10 | 10/10 |
|  | Focal inflammation |  | 1/10 | - |  | - | - |
| *Digestive system* |  |  |  |  |  |  |  |
| Tongue/larynx | Normal |  | 10/10 | 10/10 |  | 10/10 | 10/10 |
| Salivary glands | Normal |  | 10/10 | 10/10 |  | 10/10 | 10/10 |
| Esophagus | Normal |  | 10/10 | 10/10 |  | 10/10 | 10/10 |
| Stomach | Normal |  | 10/10 | 10/10 |  | 10/10 | 10/10 |
| Duodenum | Normal |  | 10/10 | 10/10 |  | 10/10 | 10/10 |
| Jejunum | Normal |  | 10/10 | 10/10 |  | 10/10 | 10/10 |
| Ileum | Normal |  | 10/10 | 10/10 |  | 10/10 | 10/10 |
| Cecum | Normal |  | 10/10 | 10/10 |  | 10/10 | 10/10 |
| Colon | Normal |  | 10/10 | 10/10 |  | 10/10 | 10/10 |
| Rectum | Normal |  | 10/10 | 10/10 |  | 10/10 | 10/10 |
| Liver | Normal |  | 1/10 | 4/10 |  | 0/10 | 1/10 |
|  | Focal inflammation |  | 9/10 | 6/10 |  | 10/10 | 9/10 |
| Pancreas | Normal |  | 10/10 | 10/10 |  | 10/10 | 10/10 |
| *Immune system* |  |  |  |  |  |  |  |
| Thymus | Normal |  | 10/10 | 10/10 |  | 10/10 | 10/10 |
| Spleen | Normal |  | 10/10 | 10/10 |  | 10/10 | 10/10 |
| Cervical lymph node | Normal |  | 10/10 | 10/10 |  | 10/10 | 10/10 |
| Mesenteric lymph node | Normal |  | 10/10 | 10/10 |  | 10/10 | 0/10 |
| *Endocrine system* |  |  |  |  |  |  |  |
| Pituitary gland | Normal |  | 10/10 | 10/10 |  | 10/10 | 10/10 |
| Thyroid glands/ Parathyroid glands | Normal |  | 7/7* | 9/9* |  | 9/9* | 8/8* |
| Adrenal glands | Normal |  | 10/10 | 10/10 |  | 10/10 | 10/10 |
| *; Total number of organs prepared and examined is shown. | |  |  |  |  |  |  |

| Supplementary Table 3. Histopathological findings in major organs from SD rats orally treated with *Acer tegmentosum* water extract for 90 days (continued from the previous page) | | | | | | | |
| --- | --- | --- | --- | --- | --- | --- | --- |
| Organ | Findings |  | Dose of *Acer tegmentosum* extract (mg/kg) | | | | |
|  |  |  | Male  (n=10/group) | |  | Female (n=10/group) | |
|  |  |  | 0 | 5000 |  | 0 | 5000 |
| *Respiratory system* |  |  |  |  |  |  |  |
| Nasal cavity | Normal |  | 10/10 | 10/10 |  | 10/10 | 10/10 |
| Trachea | Normal |  | 10/10 | 10/10 |  | 10/10 | 10/10 |
| Lung | Normal |  | 7/10 | 10/10 |  | 10/10 | 10/10 |
|  | Focal inflammation |  | 3/10 | 0/10 |  | 0/10 | 0/10 |
| *Cardiovascular system* | |  |  |  |  |  |  |
| Heart | Normal |  | 10/10 | 10/10 |  | 9/10 | 10/10 |
|  | Focal inflammation |  | 0/10 | 0/10 |  | 1/10 | 0/10 |
| *Urinary system* |  |  |  |  |  |  |  |
| Kidneys | Normal |  | 8/10 | 9/10 |  | 7/10 | 7/10 |
|  | Focal inflammation |  | 2/10 | 1/10 |  | 0/10 | 1/10 |
|  | Dystrophic calcification |  | 1/10 | 0/10 |  | 3/10 | 2/10 |
| Urinary bladder | Normal |  | 10/10 | 10/10 |  | 10/10 | 10/10 |
| *Reproductive system* | |  |  |  |  |  |  |
| Preputial gland /Clitoral glands | Normal |  | 10/10 | 10/10 |  | 10/10 | 9/10 |
| Testes | Normal |  | 10/10 | 10/10 |  | - | - |
| Epididymides | Normal |  | 10/10 | 10/10 |  | - | - |
| Prostate | Normal |  | 9/10 | 10/10 |  | - | - |
|  | Abscess |  | 1/10 | 0/10 |  | - | - |
| Seminal vesicle | Normal |  | 10/10 | 10/10 |  | - | - |
| Ovaries | Normal |  | - | - |  | 10/10 | 10/10 |
| Uterus | Normal |  | - | - |  | 10/10 | 10/10 |
| Vagina | Normal |  | - | - |  | 10/10 | 10/10 |
| *Musculoskeletal and other systems* | |  |  |  |  |  |  |
| Skin/mammary gland | Normal |  | 10/10 | 10/10 |  | 10/10 | 10/10 |
| Skeletal muscle | Normal |  | 10/10 | 10/10 |  | 10/10 | 10/10 |
| Sternum | Normal |  | 10/10 | 10/10 |  | 10/10 | 10/10 |
| Femur / bone marrow | Normal |  | 10/10 | 10/10 |  | 10/10 | 10/10 |
